# Comb-Tipped Coupled Cantilever Sensor for Enhanced Real-Time Detection of *E. coli* Bacteria

**DOI:** 10.3390/s25134145

**Published:** 2025-07-03

**Authors:** Syed Ali Raza Bukhari, Elham Alaei, Zongchao Jia, Yongjun Lai

**Affiliations:** 1 Department of Mechanical and Materials Engineering, Queen’s University, Kingston, ON K7L 3N6, Canada; 22sarb@queensu.ca (S.A.R.B.); 22ea18@queensu.ca (E.A.); 2 Department of Biomedical and Molecular Sciences, Queen’s University, Kingston, ON K7L 3N6, Canada; jia@queensu.ca

**Keywords:** gap method, cantilever sensor, MEMS, dielectrophoresis, *E. coli*

## Abstract

The detection of particulate matter, particularly pathogenic bacteria, is essential in environmental monitoring, food safety, and clinical diagnostics. Among the various sensing techniques used, cantilever-based sensors offer a promising platform for label-free, real-time detection due to their high sensitivity. Here, we present a coupled cantilever sensor incorporating interdigitated comb-shaped structures to enhance dielectrophoretic (DEP) capture of *Escherichia coli* in liquid samples. During operation, one cantilever is externally actuated and the other oscillates passively through fluid-mediated coupling. The sensor was experimentally evaluated across a broad concentration range from 10 to 10^5^ cells/mL and the resonant frequency shifts were recorded for both beams. The results showed a strong linear frequency shift across all tested concentrations, without saturation. This demonstrates the sensor’s ability to detect both trace and high bacterial loads without needing recalibration. High frequency shifts of 4863 Hz were recorded for 10^5^ cells/mL and 225 Hz for the lowest concentration of 10 cells/mL, giving a limit of detection of 10 cells/mL. The sensor also showed a higher signal to noise ratio of 265.7 compared to previously reported designs. These findings showed that the enhanced sensor design enables sensitive, linear, and reliable bioparticle detection across a wide range, making it suitable for diverse applications.

## 1. Introduction

Particulate matter (PM) sensing is essential for assessing air and water quality, with significant relevance to public health, industrial safety, and biomedical fields. Exposure to ultrafine particles (UFPs) has been associated with respiratory [1], cardiovascular [2], and neurodegenerative diseases [3], highlighting the urgent need for precise and dependable detection methods. Effective detection and characterization of these particles can allow researchers, healthcare professionals, and regulatory bodies to identify pollution sources, implement mitigation strategies, and enforce environmental standards. Detection of bioparticles is a specialized form of particulate matter detection, focused on identifying biological particles such as bacteria, viruses, and biomolecules in air and liquid media. It serves as a vital tool for food safety, environmental monitoring, and public health by enabling accurate detection of biological contaminants. Traditional detection methods such as culture-based assays and polymerase chain reaction (PCR), are highly specific but often time-consuming, labor-intensive, and dependent on complex instrumentation, limiting their utility for real-time analysis [4,5]. Sensors capable of real-time detection of particles offer a promising solution to these limitations, allowing for direct, rapid, and label-free measurement of bioparticles through physical or chemical interactions.

Due to their broad applicability, such sensors have become indispensable tools in modern analytical science. They enable fast, sensitive detection of biological analytes across a wide range of domains, including healthcare [6], environmental safety [7,8], food quality monitoring [9,10,11], and biodefense [12]. A variety of sensing technologies have been developed to address the shortcomings of conventional techniques, employing detection principles based on optical [13], electrochemical [14], and mechanical [15] mechanisms to achieve high specificity and sensitivity. Among these, mechanical bioparticle detectors and, particularly, cantilever-based systems have been widely recognized for their high accuracy, exceptional sensitivity, and potential for miniaturization [16,17]. Cantilever sensors are among the simplest and most versatile mechanical platforms used in bioparticle detection, owing to their compactness, scalability, and high sensitivity to minute physical changes [18]. These sensors operate in either static mode, where cantilever deflection is measured under applied perturbation, or dynamic mode, where shifts in resonant frequency are used to quantify changes in mass. The dynamic mode is particularly advantageous for detecting microparticles due to its higher sensitivity, and this approach has been used in various fields including particulate matter sensing [19], airborne particle detection [20], and gas sensing [21].

Cantilever-based bioparticle detectors have demonstrated significant impact across various applications, including the detection of cancer biomarkers [21,22,23], DNA [24,25], viruses [26,27], drug compounds [28], oligonucleotides [29], and a wide range of pathogens. A common approach in using such sensors involves surface functionalization of the cantilever using antibodies or other capturing agents that bind specifically to target particles, resulting in mass loading and a corresponding shift in resonant frequency. This technique has been successfully applied to detect SARS virus [30], tuberculosis biomarkers [31], cardiac markers [32], cancer-related proteins [33,34], and bacteria such as *E. coli* and *P. aeruginosa* [35]. Alternatively, cantilever-based sensors can operate without surface functionalization using techniques such as dielectrophoresis (DEP) [36], which employs non-uniform electric fields to manipulate and concentrate particles directly onto the cantilever surface. This label-free method is effective for capturing a wide range of biological targets, including *E. coli* and *P. aeruginosa*, making it ideal for applications requiring flexible and real-time manipulation of particles.

A well-established DEP-based sensing technique is the gap method, which is traditionally implemented using a single cantilever beam positioned adjacent to a fixed wall [37]. A narrow micron-sized gap between the free end of the cantilever and the wall serves as the region where DEP is applied to trap the bioparticles. Under the influence of DEP, particles move towards the gap and attach to the cantilever surface near the tip. The accumulation of these particles alters the cantilever’s resonant frequency, due to the increased mass from the particles. However, another key contributor to the frequency shift is the pearl chain effect, where particles align to form chains across the gap in the form of streamers, significantly increasing the damping experienced by the cantilever. The combined influence of mass loading and increased damping results in a pronounced frequency shift, enabling improved sensitivity, especially at low concentrations. Previous studies have demonstrated the utility of this technique for detecting a variety of bioparticles, including *E. coli*, *P. aeruginosa*, and other pathogens [38].

Despite its effectiveness, the traditional gap method has a key limitation, that is, only the particles captured on the cantilever contribute directly to the frequency shift. A significant portion of the biological load accumulates on the fixed wall, which does not oscillate and thus does not directly influence the measured resonant frequency. This partial detection can lead to underestimation of particle concentration and limit the sensor’s quantitative accuracy. To address this limitation, an alternate approach has been introduced, in which the fixed wall is replaced by a second cantilever beam. DEP is applied across the gap between the two freely oscillating beams, allowing particles to be captured on both surfaces. Since both beams participate in the dynamic response, the entire captured bacterial mass contributes to measurable frequency shifts. This configuration not only improves sensitivity but also ensures a more accurate representation of particle loading. Additionally, the presence of two beams enhances the fluidic coupling within the gap, which can lead to beneficial dynamic effects such as quality factor enhancement in the driven beam [39,40].

This paper presents a coupled cantilever sensor with interdigitated protrusions at the tip of the cantilever. The sensor utilizes the gap method and DEP to capture E. coli bacteria in the interdigitated protrusions between the two cantilever beams. In this configuration, only one of the cantilever beams (driving beam) is directly excited using an external signal. The second beam, known as the driven beam, is not externally actuated, it oscillates under due to the hydrodynamic coupling with the surrounding fluid. This passive oscillation results in a significant reduction in the damping and an increase in the quality factor of up to twice that of the driven beam, which improves the sensitivity and limit of detection of the sensor. This approach addresses the shortcomings of previously reported single beam sensors by accounting for the complete captured bacterial load as the frequency shift for both cantilever beams is measured. Moreover, the addition of interdigitated comb-shaped protrusions at the cantilever tips further enhances the sensing capability by significantly increasing the particle capture area. The sensitivity, linearity, and dynamic range of the sensor are also positively affected by the design modifications; detailed experimental analysis is conducted to quantify the improvements in the performance of the sensor.

## 2. Materials and Methods

### 2.1. Design and Fabrication

The coupled cantilever sensor reported here was designed following the constraints of the commercially available multiuser MEMS (microelectromechanical systems) process of PiezoMUMPs, which is offered by the Science Foundry, Durham, NC, USA [41]. In addition to being a mature fabrication process, PiezoMUMPs is capable of fabricating high aspect ratio microstructures with minimum air damping, due to absence of substrates below the moving microstructure. It provides a platform that integrates piezoelectric thin films into MEMS devices and allows for the fabrication of devices requiring piezoelectric and piezoresistive properties. The devices made using this process consist of five layers, a 1.02 µm Aluminum and Chromium metal top layer for the contact pads, followed by a 0.5 µm Aluminum Nitride piezoelectric layer, a silicon device layer with a thickness of 10 µm, a 1 µm thick oxide layer, and finally a silicon substrate with a thickness of 400 µm.

**Figure 1:**
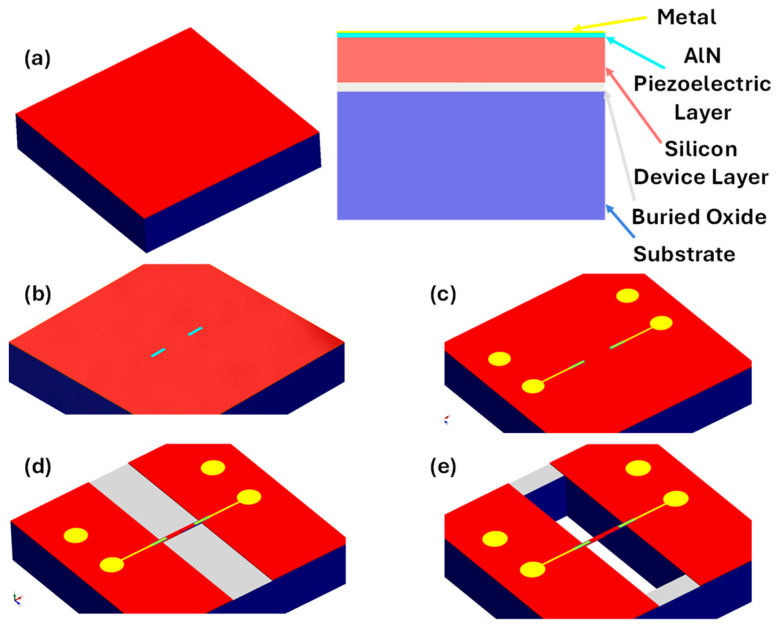
Fabrication process steps for PiezoMUMPS. (**a**) SOI Wafer. (**b**) Piezoelectric layer deposition. (**c**) Metal layer deposition. (**d**) Deep Reactive Ion Etching (DRIE) of the Silicon device layer. (**e**) Etching of the substrate.

The PiezoMUMPS fabrication process begins with a silicon-on-insulator (SOI) wafer, which consists of a 10 µm thick silicon device layer, a 1 µm oxide layer, and a 400 µm thick silicon substrate layer. The first step involves silicon doping to modify the electrical properties of the device layer (Figure 1a). Following this, a 0.5 µm aluminum nitride piezoelectric layer is deposited via sputtering onto the silicon device layer, for piezoelectric actuation (Figure 1b). Then a top metal layer is deposited and patterned to form the electrode contact pads (Figure 1c). The MEMS structures are then defined using photolithography, and Deep Reactive Ion Etching (DRIE) is performed to etch through the silicon device layer to form the sensor as shown in Figure 1d. Afterwards, the top side of the wafer is coated with a protective layer and the wafer is turned over to lithographically pattern it from the bottom side. A DRIE silicon etch is subsequently used to etch these features completely through the substrate layer followed by wet etching of the oxide layer to completely release the device as shown in Figure 1e.

**Figure 2:**
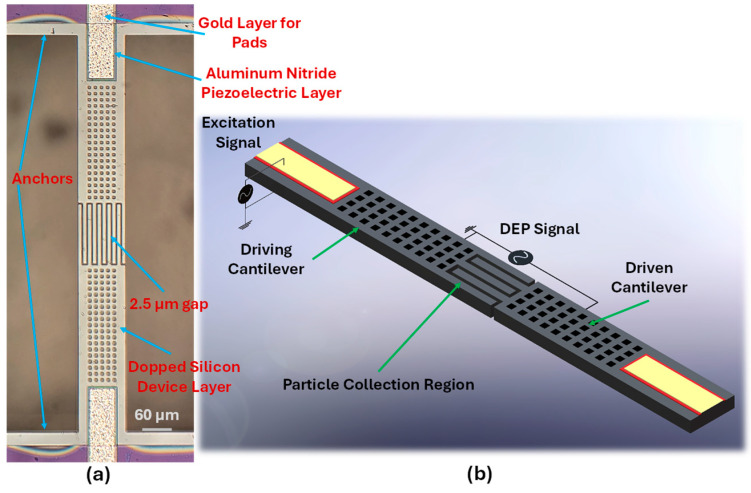
Design of the proposed comb-tipped coupled cantilever sensor (**a**) Microscopic image of the fabricated sensor. (**b**) Schematic showing the different parts of the sensor and the voltage application methodology.

A schematic of the proposed sensor is shown in Figure 2b, the sensor consists of a pair of cantilever beams with comb-shaped interdigitated protrusions at the tip which have a gap of approximately 2.5 µm between them. Both cantilever beams have an overall length of 375 µm, while the protrusions at the end have a length of 100 µm. The width of each beam is 78 µm and the width of the protrusions at the tip of the cantilevers is 6 µm. The design utilizes these interdigitated comb-shaped structures to increase the total surface area available for the collection of particles. Both cantilever beams have holes which go through the entirety of the structure, these holes are made to further reduce the effect of damping experienced by the cantilevers due to their out of plane actuation. The cantilever beams are actuated using a piezoelectric actuation layer deposited on top, this layer has a width of 50 µm and a length of 80 µm. The metal layer on top is deposited over the piezoelectric layer for the application of the voltages. The final fabricated sensor is shown in Figure 2a.

### 2.2. Operation of the Device

The proposed sensor utilizes the gap-method for the detection of *E. coli*. In contrast to the traditional configuration for the gap method, where a single cantilever beam is facing a fixed wall with a micron sized gap between the two beams used to collect the particles, the sensor reported here replaces the fixed wall with a second cantilever beam. The use of a second cantilever beam instead of a fixed structure allows for a symmetric system where all collection surfaces contribute to the output of the sensor. Moreover, the design also incorporates comb-shaped interdigitated protrusions at the tips of both cantilevers. These structures increase the sensitive region by a substantial amount which results in a higher collection capacity for the sensor. In this design, the DEP is applied across the two beams, concentrating *E. coli* cells into the gap region between the comb-shaped protrusions, where the electric field gradient is strongest. During operation, only one cantilever (referred to as the driving beam) is actively actuated by an external signal, while the second beam (the driven beam) oscillates passively through coupling with the surrounding fluid. Due to the fluid-driven motion of the driven beam, it faces less damping and has a higher quality factor which results in a lower noise level and higher sensitivity.

**Figure 3:**
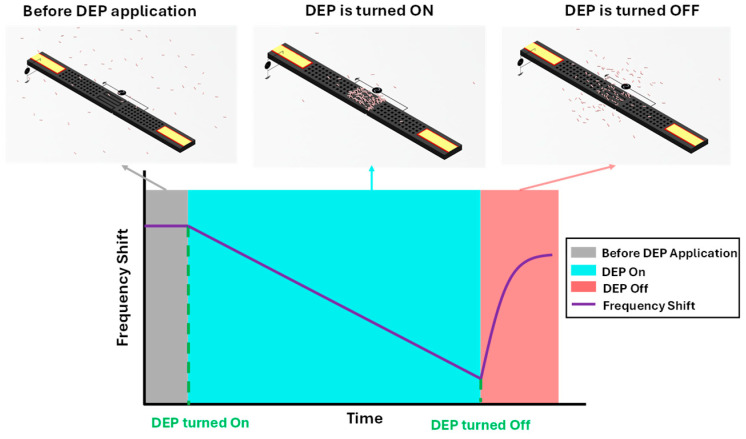
Schematic representation of the coupled cantilever sensor operating in stagnant samples, illustrating key stages of sensor function. Initially, bioparticles are randomly distributed with minimal impact on frequency (**top left**). Upon application of DEP, particles begin to accumulate in the gap region, resulting in a frequency decrease (**top center**). After DEP is turned off, some particles disperse, leading to a partial recovery of the resonant frequency (**top right**).

The operation of the sensor is schematically shown in Figure 3. Initially, there is no DEP voltage applied, so there is no change in the resonant frequency of the cantilevers, but once the voltage is applied, the bacteria start accumulating within the gap between the interdigitated protrusions and along the surfaces of both beams. The presence of these particles induces a shift in the resonance frequencies of both cantilevers. However, as discussed before, the frequency change is not merely due to the added mass from the particles and there is a second phenomenon called the pearl chain effect which is inducing an additional frequency shift. The pearl chain effect is the formation of chains of the captured particles, these particles loosely attach to each other and to the comb-shaped protrusions in the gap region. These chains of particles do not bridge the gap, but rather they act similar to the dendrites formed on electrodes [42]. These pearl chains of particles act as streamers and cause a significant increase in the damping which results in a much higher shift in the resonant frequency. Moreover, as both cantilevers participate in the detection process, the full bacterial load trapped in the gap region contributes to the frequency response. This offers a significant advantage over single-beam sensors, where only one oscillating surface is sensitive, while the fixed wall only contributes partially due to the presence of streamers and does not influence the frequency directly. Additionally, the particles accumulating on the surface of the wall farther from the gap region in the single beam systems do not contribute to the frequency shift, whereas in a coupled cantilever system, particles captured on the surfaces of both cantilevers contribute to the output. After a set amount of time of DEP application, it is turned off and the frequency starts recovering as the particles move away from the capture region as shown in the figure below. After some time, the resonant frequency value stops changing significantly and flattens out; however, this settled frequency value is lower than the initial value. The resonant frequency does not recover completely to its original value as there are some particles still stuck in the gaps between the protrusions. This does not affect the sensor permanently as the sensor recovers completely to its original state after the cleaning process which is explained in the next sections.

### 2.3. Experimetal Setup and Methodology

#### 2.3.1. Experimental Setup

**Figure 4:**
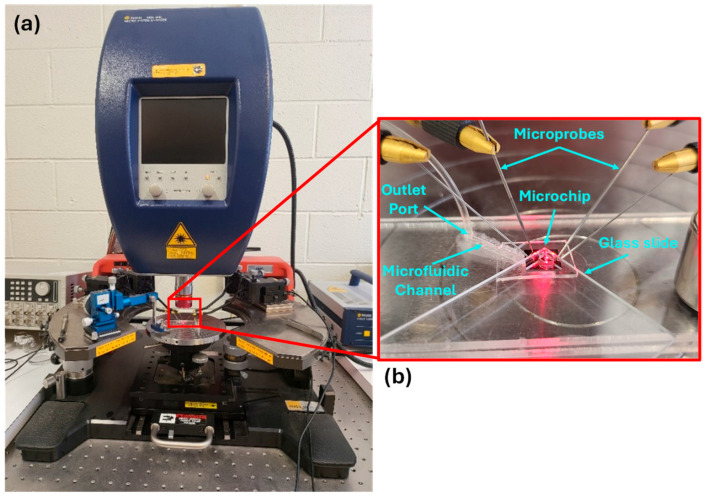
Experimental setup. (**a**) Polytec Scanning Vibrometer MSA-400. (**b**) Microfluidic platform with the fabricated microchip on top of the wafer chuck of MSA-400.

The experimental setup for characterizing the coupled cantilever sensor is shown in Figure 4. The microchip with the coupled cantilever sensor is mounted on a PMMA-based microfluidic platform (Figure 4b), the surface of the PMMA plate is machined to make a 1.5 mm-wide channel. Another PMMA plate is then used as the top layer to form a sealed channel, then two holes are drilled through the top plate to make the inlet and outlet ports. Afterwards, the setup is placed on the wafer chuck of a Polytec MSA-400 (PolyTec, Irvine, CA, USA) vibrometer and a magnet is used to hold the platform firmly (Figure 4a). The microchip is placed over one of the ports and a 150–200 μL droplet of the sample solution is then placed over the microchip, furthermore, a syringe connected to the other port is used to draw the droplet into the channel to submerge the sensor completely. Afterwards, a glass slide mounted on a micro-positioner is used to flatten the droplet to allow for clear viewing using a microscope. For the application of voltages, four microprobes which are held by micro positioners are utilized; two of these are connected to the built-in signal generator of the vibrometer for applying the actuation voltages, whereas the remaining two probes are connected to an external signal generator (Agilent 33220A; Agilent, Santa Clara, CA, USA) and are used to apply the voltages for DEP.

#### 2.3.2. Preparation of Samples

For experimental characterization of the device, the samples used were either deionized (DI) water or a particular concentration of E. coli bacteria in DI water. Initially, a sample of live *E. coli* bacteria (K-12) was prepared, and the concentration was determined using a spectrophotometer. This initial sample had a concentration of 10^8^ cells/mL which was then serially diluted by adding 10% concentrate and 90% DI water to generate the required test concentrations. Samples ranging from concentrations as high as 10^5^ cells/mL and as low as 10 cells/mL were prepared using this method. Each diluted sample was then mixed thoroughly using a vortex mixer (SI-T236; Scientific Industries, New York, NY, USA) for two minutes to ensure even distribution of the bacterial cells throughout the liquid prior to testing. Moreover, before using these bacterial suspensions for the experiments, they were exposed to ultraviolet light (UV-B), delivering a total dose of approximately 3 mJ/cm^2^. This treatment effectively deactivates the bacteria ensuring that they are no longer capable of replication [43].

#### 2.3.3. Experimental Methodology

For characterizing the dynamic response of the device, a sample consisting only of DI water is used. A droplet of DI water is placed on the chip and flattened using the glass slide. Afterwards, a 3 V_p_ actuation voltage with a frequency sweep from 50 kHz to 1 MHz is applied using the built-in signal generator in the MSA-400, and the response is recorded from both cantilever beams. For characterizing the performance of the sensor in different concentration samples, the water droplet is replaced with a sample containing E. coli (10–10^5^ cells/mL). In addition to actuation voltage, a 4 V signal at 200 kHz is applied for the DEP between the driving and driven cantilever using the external signal generator. For higher concentration samples, these experiments last 7 min, with DEP activated at the 1 min mark and deactivated at the 6 min mark, after which the frequency is allowed to recover for one minute, giving a total particle collection time of 5 min. The resonant frequency is then automatically recorded using a python script along with the Polytec software, PSV 9.3 which measures the frequency value at set intervals. For the low concentration experiments, longer application of DEP is necessary to observe a substantial frequency shift, therefore the experiments are 32 min long and the DEP is applied for 30 min.

For the gap-method-based sensors, all the particles captured in the gap region are not released after the deactivation of DEP and some particles remain stuck in the gap region or on the surface of the cantilevers and can cause measurement errors in the subsequent experiments. To avoid this and ensure that there are no residual bacteria affecting the data in subsequent experiments, between experiments the microchip is heated to 450 °C on a hotplate (Corning PC-400D; Corning, New York, NY, USA) for 10 min to vaporize residual biological material [44]. Afterwards, the device is allowed to cool down to room temperature and the frequency response is recorded to ensure that the sensor returns to original conditions.

## 3. Results

### 3.1. Dynamic Response of the Sensor

**Figure 5:**
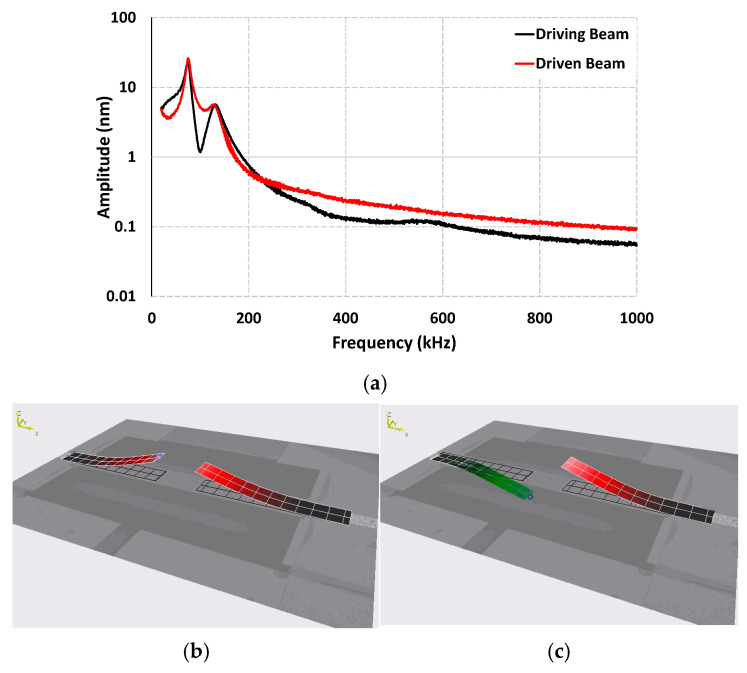
(**a**) Frequency response of the comb-tipped coupled cantilever sensor in water, obtained using the MSA-400 vibrometer. Mode shapes of the sensor: (**b**) first mode with in-phase oscillation and (**c**) second mode with out-of-phase oscillations.

The dynamic response of the coupled cantilever sensor was first characterized in a liquid environment before introducing biological particles. The frequency response for both the driving and driven beams was measured using the MSA-400 vibrometer, as shown in Figure 5a. The results indicate that the driven beam has a higher quality factor compared to the driving beam. The driving beam has a quality factor of 6.1, while the driven beam achieved a value of 9.7, which is significantly higher. This improvement can be attributed to the passive oscillation of the driven beam, which has induced oscillations due to the surrounding medium and experiences reduced damping compared to the actively excited driving beam. As discussed previously, this passive behavior results in a sharper resonance peak for the driven beam, leading to lower noise and enhanced sensitivity. The mode shapes for the sensor were also obtained using the vibrometer, and the results are shown in Figure 5b,c. Two distinct vibration modes were identified: in the first mode, both cantilever beams oscillate in phase, moving synchronously with each other; in the second mode, the beams oscillate out of phase. Animations of the vibrational modes for the sensor obtained from the Polytec vibrometer software are provided in the Supplementary Materials.

**Figure 6:**
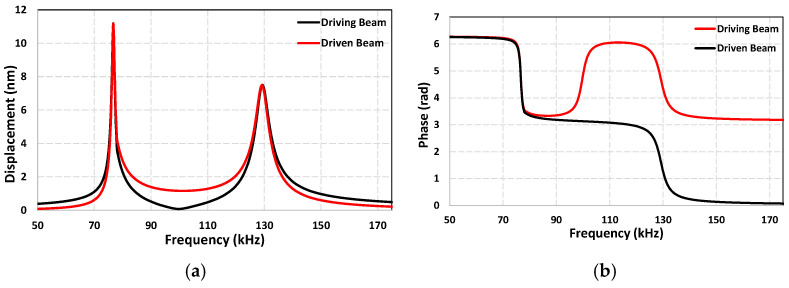
(**a**) Frequency response for both cantilever beams obtained using COMSOL Multiphysics. (**b**) Phase response.

In our previously developed coupled cantilever sensor with a simple beam geometry and without any protrusions at the tips, two primary vibrational modes were observed as well. The first mode with out-of-phase oscillation, and the second mode with in-phase oscillation. A doubling of the quality factor was observed for the driven beam in the first mode, which was suspected to be the result of the driven beam being oscillated passively under the influence of the surrounding medium and thus experiencing less damping compared to the driving beam. However, for the current sensor design, this modal behavior undergoes a notable shift. The first mode now corresponds to in-phase oscillation whereas the second mode is out of phase, which is the opposite of the previously reported sensor. The doubling of the quality factor is still observed in the first mode, which is now the in-phase mode instead of the out-of-phase mode. This inversion in modal behavior of the sensor suggests that the introduction of the comb structures fundamentally altered the coupling dynamics and energy distribution between the cantilever beams. To better understand this phenomenon, we performed a detailed fluid–structure interaction simulations using COMSOL Multiphysics 6.1. The model included both cantilevers fully submerged in water, with the same material properties and geometry as the fabricated device. The simulation results shown in Figure 6 confirmed the experimental observations, the results showed that the first mode corresponds to in-phase oscillation of the two beams, and the second mode is out of phase.

One plausible explanation for this behavior is that the presence of interdigitated structures at the beam tips results in stronger mechanical coupling between the two cantilevers as the extended surface area and closely spaced protrusions increase the interaction between the two beams through the fluid. This increased coupling strength may cause the coupled system to favor the in-phase mode instead of the out-of-phase mode; the use of coupling strength to alter the modal behavior of the sensor has been reported in various studies [45,46,47]. We also suspect that the comb structures may provide a more synchronized movement of the fluid and the beams in the in-phase mode, reducing relative shear and fluid drag and therefore enhancing the quality factor of the driven beam in this mode. Conversely, the second mode may involve a more conflicting fluid dynamic behavior around the interdigitated tips, resulting in higher damping and thus lower quality factor values. Considering this, the sensor presented here was operated in the first mode due to a higher quality factor and the stable nature of the first mode.

### 3.2. Frequency Shifts at Different Concentrations of E. coli

**Figure 7:**
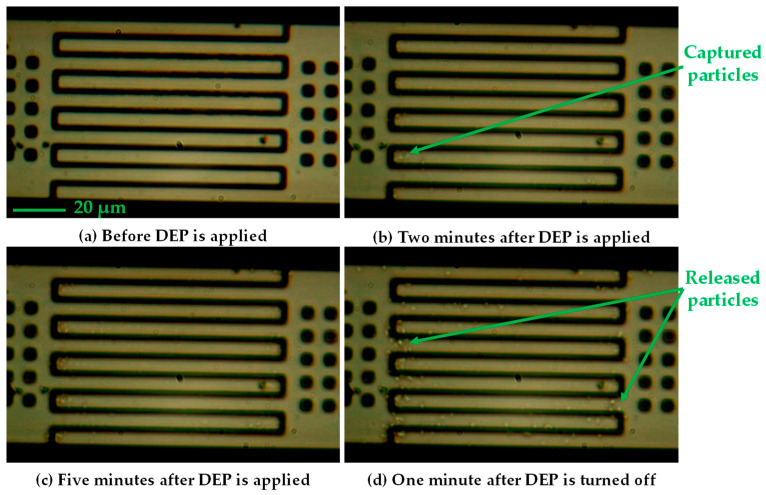
State of the sensor at different times during the operation.

To characterize the performance of the coupled cantilever sensor, multiple experiments were performed using *E. coli* samples at different concentrations. To observe the behavior of the bioparticle detector during operation, the device was set up under a microscope and a sample with a particle concentration of 10^4^ cells/mL was used. The operation of the sensor can be observed pictorially in the sequential images captured during different stages of DEP application as shown in Figure 7; a video of this experiment is also provided in the Supplementary Material. This experiment was performed using a DEP voltage of 4 V while no actuation voltage was applied. The images show that prior to the activation of the DEP signal, no bacteria accumulation is visible within the gap or on the comb fingers (Figure 7a). After the application of the DEP, the bacteria start accumulating in the gap region between the comb-shaped protrusions and after 2 min, some bacteria are observed next to the gaps and on the surface of the cantilevers (Figure 7b). With a longer application time for the DEP, there is a significant particle accumulation and after 5 min of DEP application, a much higher concentration of bacteria can be seen along the gap region and the surfaces of the protrusions (Figure 7c). At the 6 min mark the DEP signal is turned off, and an image captured 1 min after deactivation shows dispersal of the bacteria (Figure 7d). However, as discussed before, a considerable number of the particles remain attached to the comb structures and in the gap, leading to a partial recovery of the resonant frequency rather than a full return to the original value which are then removed using thermal ablation.

**Figure 8:**
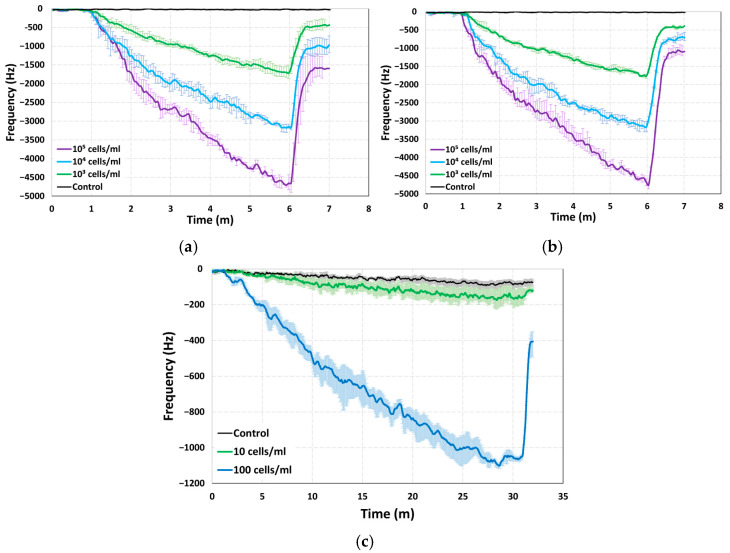
Frequency shift response of the comb-tipped coupled cantilever sensor from experiments with samples containing different concentrations of *E. coli* and control samples. For the higher concentrations of 10^3^, 10^4^, and 10^5^ cells/mL: driving beam response (**a**) and driven beam response (**b**). The response at low concentrations of 10 and 100 cells/mL along with a control experiment over a longer time period (**c**).

The performance of the proposed coupled cantilever sensor was also evaluated by monitoring the resonant frequency shifts during particle collection across varying concentrations of *E. coli* bacteria (10 to 10^5^ cells/mL) and control samples of pure deionized water. For each concentration, three independent trials were performed, and the average frequency shifts were reported to account for run-to-run variations. The frequency shift responses for the different concentrations are shown in Figure 8. During the first minute of operation without the application of DEP, the resonant frequency remains stable, confirming that no bacteria are being collected. Upon activation of the 4 V DEP signal at the one-minute mark, a noticeable and continuous decline in the resonant frequency is observed as bacteria are captured in the gap region and on the surface of the comb-shaped protrusions of the cantilevers. For the concentrations of 10^3^–10^5^ cells/mL, the DEP remains active until the six-minute mark, allowing sufficient time for significant bacterial accumulation. After turning off the DEP, a partial recovery in resonant frequency is observed since some of the particles remain attached to the surfaces and within the gaps. For the driving beam, the results from the control experiment show minimal change in the frequency value, whereas the maximum resonant frequency shifts for *E. coli* concentrations of 10^3^, 10^4^, and 10^5^ cells/mL, are recorded as 1865 Hz, 3233 Hz, and 4899 Hz, respectively, and are shown in Figure 8a. The corresponding results for the driven beam are presented in Figure 8b; the control value again shows minimal change whereas the maximum frequency shifts are 1799 Hz, 3291 Hz, and 4863 Hz, for concentrations of 10^3^, 10^4^, and 10^5^ cells/mL, respectively. These measured frequency shifts are significantly larger than those reported in previous studies for similar cantilever-based sensors, highlighting the enhanced sensitivity achieved by the modified sensor geometry. The integration of interdigitated comb-like protrusions at the cantilever tips not only increases the effective capture area but also facilitates more efficient particle trapping, leading to a stronger and more consistent frequency response.

For the lower concentration samples, the experiment time was increased to 32 min and the frequency was recorded for control samples and concentrations of 10 and 100 cells/mL. This longer total experimental time is necessary to accommodate the sparse distribution of particles in low concentration samples. While longer DEP application could potentially lead to evaporation or temperature rise in the sample due to Joule heating. These effects are minimized in our setup due to the minimal exposed surface area, the use of a low DEP voltage and frequency, and the low conductivity of the deionized water medium [48,49]. As a result, both evaporation and thermal effects are considered negligible. The results from low concentration experiments are shown in Figure 8c; they exhibit a maximum frequency shift value of 1117 Hz for 100 cells/mL and 225 Hz for 10 cell/mL. From the results, it can be seen that the sensor is capable of detecting concentrations as low as 10 cells/mL; however, the run-to-run variance between the experiments for both 100 cells/mL and 10 cells/mL concentrations is considerable. The overall shift of 1117 Hz for the concentration of 100 cell/mL is higher than the value for previously reported sensors [37,38,40]; however, compared to the results for other concentrations where the frequency shift values are up to 1.5 times higher, this increase in the frequency shift is much lower. This can be attributed to the presence of a limited number of particles in the samples at such low concentrations since even with a higher capture area for the proposed sensor, the particles available are limited and thus cannot cause a frequency shift beyond a certain amount. This is also evidenced from the plot in Figure 8c, where after the 27 min mark, the frequency shift change almost stagnates until the DEP is turned off. Moreover, for the concentration of 10 cells/mL, not all experiments yield a significant frequency shift, as the distribution of particles in the samples is not consistent; therefore, at such low concentrations, the probability of obtaining an empty sample is high. However, the repetition of the experiments yields an average value which is still statistically significant compared to the control value.

**Figure 9:**
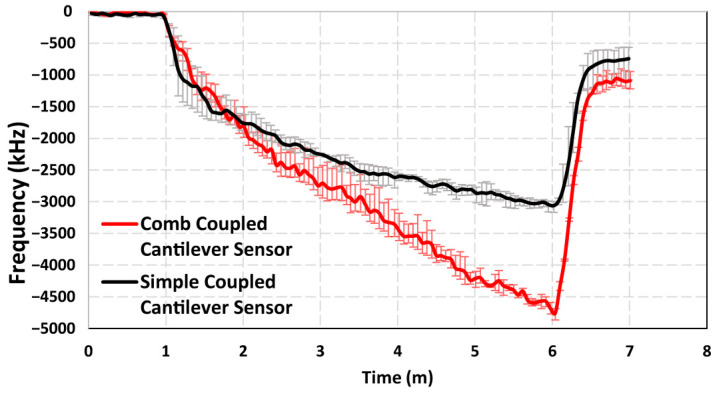
Frequency shift response at 10^5^ cells/mL concentration for the comb-tipped coupled cantilever sensor and the previously reported simple coupled cantilever sensor, depicting the difference in the linearity of the responses.

Linearity of the results is also a critical performance parameter for sensors as it reflects the sensor’s ability to provide accurate and predictable responses across varying analyte concentrations. An important outcome of this study is the observation of a highly linear correlation between the measured resonant frequency shift and the concentration of *E. coli* across the tested range of 10 to 10^5^ cells/mL. Unlike prior sensor designs that exhibited saturation effects at higher concentrations, which limits their detection range, the current device maintains a highly linear relationship between frequency shift and bacterial concentration across the tested range. The proposed sensor utilizes interdigitated comb-shaped protrusions at the free ends of both cantilever beams, which provide a significantly enlarged and spatially distributed area for particle collection. This design enhancement ensures that the particles are distributed more evenly and have a large area available for collection, which mitigates the possibility of saturation at the tested concentrations. A comparison of the results for 10^5^ cells/mL concentration from our previously reported sensor with a simple beam geometry with the current design is shown in Figure 9. The results show that the new design shows a much more linear response and a consistent frequency shift throughout the experiment compared to the previously reported sensor which shows a sharper frequency shift in the beginning of the experiment and then starts to become more saturated as time goes on. For the particle collection period between the 1 min and 6 min mark, the comb-tipped coupled cantilever design achieved a coefficient of determination (R^2^) value of 0.97 at this concentration, while the simple coupled cantilever design saturates at higher bacterial loads and shows an R^2^ value of 0.84. This linearity of the results further emphasizes the advantages of the redesigned capture region and indicates the sensor’s potential for reliable detection over a broader range of bacterial concentrations.

### 3.3. Limit of Detection, Signal to Noise Ratio, and Dynamic Range of the Sensor

**Figure 10:**
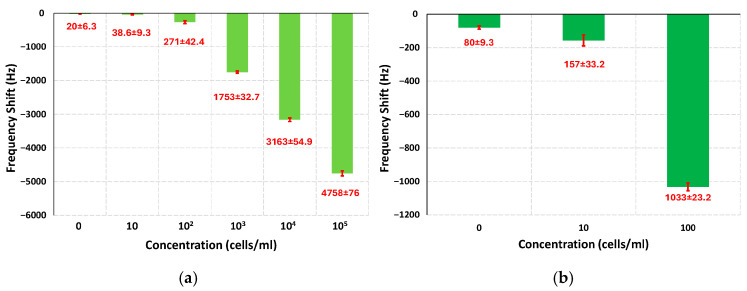
Average frequency shift and the standard deviation values for the different concentrations of *E. coli* and control samples. (**a**) Data from experiments for all concentration samples at the 6 min mark just before the DEP is turned off. (**b**) Data from the low concentration samples of 10 cells/mL, 100 cells/mL, and control samples, at the 31 min mark just before the Dep is turned off.

The limit of detection (LOD) is an important parameter for characterizing the performance of a sensor, it represents the lowest detectable concentration of the analyte that yields a statistically distinguishable change in the resonant frequency relative to the baseline value. LOD can be estimated theoretically using the effective mass and quality factor of the cantilever system if the resonant frequency shift is assumed to arise solely from added particle mass [17,50]. However, in gap-method-based sensors, in addition to the mass, the increased damping resulting from the formation of pearl chains in the gap region also plays a major role. This added damping contributes substantially to the overall frequency shift and must be accounted for in any realistic LOD estimation. Therefore, a more practical and accurate estimation of the LOD can be obtained using the method described in [50], which defines LOD as the analyte concentration corresponding to a frequency shift equal to the minimum observed shift (control value) plus three times the standard deviation of that measurement. Figure 10 shows the frequency shift values for the different concentrations tested. For the results shown in Figure 10a, the frequency shift value is taken at the 6 min mark after the DEP was applied for 5 min. The average frequency shift value for the control experiment is 20 with a standard deviation value of 6.3, which gives a statistically significant frequency shift value of 38.9 Hz. This value is higher than the value for 10 cells/mL concentration but much lower than the rest of the concentrations and shows that for the short time experiments, the sensor is unable to reliably measure the bacterial load at extremely low concentrations.

However, if the time for the experiments is increased, the frequency shift values increase as well since more time is available for the collection of particles. Figure 10b shows the average frequency shift and standard deviation values for the 32 min-long experiment for the control value as well as the 10 and 100 cells/mL concentrations. With the minimum value of 80 Hz and a standard deviation of 9.3, the statistically significant frequency value is 107.9 Hz, which is lower than the average value obtained for the 10 cells/mL concentration. Therefore, with a statistically significant signal at 10 cells/mL concentration, the LOD for this sensor can be considered as around 10 cells/mL. This represents a notable improvement over previously reported gap-method-based sensor designs and can be attributed to the design enhancement of the comb-shaped protrusions. These geometric features allow for a larger area for the interaction of the particles with DEP thus increasing the chance of capturing the particles. The sensitivity value of the sensor is also an important parameter and can be calculated using the formula $\sigma =f_{res}/2\times m_{eff}\;$, where *m_eff_* is the effective mass of the cantilever and *f_res_* is the resonant frequency [17]. The experimentally measured frequency values were 72.8 kHz and 75.4 kHz for the driving and driven beam, respectively; using the stiffness value for the beams and these resonant frequency values, the effective mass values were calculated to be 11.8 ng and 11 ng. Using these values the sensitivity was found to be 3.08 Hz/pg and 3.43 Hz/pg for the driving and driven beam, respectively. These sensitivity values are calculated considering the effect of mass which is not the only factor impacting the performance of the sensor as previously discussed; the damping due to formation of pearl chains has a drastic effect on the output of the sensor as well. Therefore, the actual sensitivity values for the sensor are likely to be much better.

In addition to a low limit of detection, the sensor demonstrates a high signal-to-noise ratio (SNR), which is a critical parameter for sensors. The SNR is calculated by dividing the resonant frequency shift obtained just before the DEP is deactivated, by the root mean square (RMS) noise of the control measurement. The RMS noise for the control experiment for the low concentrations of 10 cell/mL and 100 cells/mL, is 56.4, with the frequency shift values of 225 Hz and 1117 Hz the SNR values are 4 and 19.8, respectively. For the higher concentrations (10^3^, 10^4^ and 10^5^) the RMS value was measured to be approximately 18.3 Hz, using this value and the driven beam frequency shift values of 1799 Hz, 3291 Hz, and 4863 Hz, for concentrations of 10^3^, 10^4^, and 10^5^ cells/mL, respectively, the SNR values were found to be 98.3, 180.2, and 265.7, respectively. These values are substantially higher than those reported for previous gap-method-based sensors utilizing single as well as coupled cantilevers since the highest value among these for the 10^3^ cells/mL concentration are 17 to 41.2 [40]. The enhanced SNR can be attributed to the significantly increased capture area provided by the interdigitated tips of the cantilevers.

Another important performance metric is the dynamic range of the sensor, which refers to the span between its lowest detectable analyte concentration (limit of detection) and the highest concentration at which it can maintain an accurate and reliable response [50]. A wide dynamic range enables a sensor to detect both trace-level and abundant target particles without the need for repeated calibration or sample dilution. It is particularly valuable in bioparticle detection where target concentrations may vary significantly, such as air and water quality monitoring. For the proposed sensor, the dynamic range extends from 10 to 10^5^ cells/mL, covering four orders of magnitude. At the lowest tested concentration of 10 cells/mL, while the frequency shift exhibited some variability between experiments, the response remained distinguishable from the control value, whereas for the highest concentration, the response remained linear. This confirms the sensor’s capability to detect extremely low concentrations of *E. coli* as well as high concentrations. Moreover, the high end of the dynamic range can be extended further since the sensor shows a strong linear response at 10^5^ cells/mL and we suspect the sensor is capable of detecting even higher concentrations, although the response might be less linear for such high concentrations.

The results presented here demonstrate significant performance improvements over previous cantilever-based sensors utilizing the gap method. The integration of interdigitated comb-shaped protrusions at the cantilever tips has proven highly effective in enhancing the particle capture capacity, enabling stronger and more linear frequency responses at very small concentrations as well as high concentrations without saturation. Moreover, in comparison with other modern bacterial detection technologies, our cantilever-based sensor offers several advantages. Optical manipulation and detection techniques for bioparticles such as surface plasmon resonance enable label-free detection of particles and can achieve low LOD values on the order of 100 cells/mL, but often require expensive laser sources, high-precision optics, and complex operation, which increase both operational and fabrication costs [51,52,53]. While some low-cost optical sensors have also been reported in the literature, they still involve complex fabrication processes, limiting widescale application [54]. Electrochemical sensors generally provide a rapid and cost-effective method of particle detection and can offer label-free detection for low concentrations on the order of 10 cells/mL. However, they typically require surface functionalization and may suffer from issues such as electrode fouling and have a limited shelf life [55,56]. Other sensing platforms that employ magnetic manipulation of particles, combined with detection methods, such as magnetoelastic sensing or magneto-resistive sensing, can also provide rapid detection and portability along with low LOD values down to 10 cells/mL, but they often require particle labeling and complex microfluidic setups [57,58]. The reported coupled cantilever sensor, although fabricated using a relatively complex microfabrication process of PiezoMUMPs, has a very simple structure and is capable of mass production using the same fabrication process, which can result in a reduction in cost. It achieves a low LOD of 10 cells/mL without the need for surface functionalization and offers a wide dynamic range. Furthermore, the sensor demonstrates rapid response times, particularly at higher concentrations, where detection is possible within just a few minutes.

Although the reported device has some advantages compared to the other particle detection techniques discussed, it still has a capacity for further improvements. At ultra-low concentrations, the frequency shift response of the sensor is not clearly distinguishable from control values. This can be improved by using surface coatings to further enhance the capture of particles which will result in a more consistent response at low concentrations. The sensor also lacks specificity in the current state which can also be improved by using surface coatings which will allow the selective detection of a particular type of particle in samples containing different particles. Furthermore, to reduce the collection time at low concentrations, a comprehensive study for optimizing the geometry of the collection region as well as the DEP voltage while minimizing the Joule heating effect can be conducted to obtain a more optimized sensor design. Moreover, using a built-in sensing mechanism, such as using the piezoelectric layer deposited on the driven beam, to sense the passive oscillations and thus measure the resonant frequency, can allow the sensor to be used outside the lab environment without requiring bulky equipment. These improvements will be considered in future work on these types of devices. Nonetheless, even in the current state, the ability of the sensor to maintain high sensitivity values, a high signal to noise ratio and a near-linear response over a concentration range of 10 to 10^5^ cells/mL range highlights the robustness of the sensor design. This wide dynamic range, combined with a low detection limit and strong sensitivity, highlights the potential of this sensor as a sensitive, reliable, and scalable platform for real-time detection of bacterial pathogens in liquid environments.

## 4. Conclusions

This study introduces an enhanced coupled cantilever bioparticle detector incorporating interdigitated comb-shaped tip structures for the detection of Escherichia coli in liquid samples. The sensor operates using the gap method with DEP-based particle manipulation and utilizes fluid mediated coupling between two cantilever beams. This configuration addresses some of the limitations of the previous designs, ensuring that the entire bacterial load captured in the sensing region contributes to the measured frequency shift. Moreover, the addition of interdigitated protrusions at the cantilever tips significantly increases the gap region for DEP application, enabling effective and uniform particle trapping. The proposed sensor demonstrates a strong linear frequency response across a wide dynamic range of 10 cells/mL to 10^5^ cells/mL, with maximum frequency shifts reaching 4899 Hz and 4863 Hz for the driving and driven beams, respectively. The sensor also demonstrated reliable performance at lower concentrations, with a limit of detection estimated to be around 10 cells/mL. Additionally, the much higher SNR value of 98.3 at 10^3^ cells/mL concentration compared to the previously reported designs shows the improvement in signal clarity, enabling more reliable detection of particles. The proposed design offers improved capture efficiency, enhanced sensitivity, SNR and LOD as well as a more consistent performance. These attributes make it a promising tool for applications that require accurate detection of bioparticles across a wide dynamic range.

## Data Availability

Data will be made available on request.

## Supplementary Materials

The following supporting information can be downloaded at: https://www.mdpi.com/article/10.3390/s25134145/s1, Video S1: First vibrational mode (in-phase); Video S2: Second vibrational mode (out-of-phase); Video S3: Particle collection at 3.5x speed.
